# Concomitant medication use in children with autism spectrum disorder: Data from the Autism Biomarkers Consortium for Clinical Trials

**DOI:** 10.1177/13623613221121425

**Published:** 2022-09-09

**Authors:** Logan Shurtz, Chloe Schwartz, Charlotte DiStefano, James C McPartland, April R Levin, Geraldine Dawson, Natalia M Kleinhans, Susan Faja, Sara J Webb, Frederick Shic, Adam J Naples, Helen Seow, Raphael A Bernier, Katarzyna Chawarska, Catherine A Sugar, James Dziura, Damla Senturk, Megha Santhosh, Shafali S Jeste

**Affiliations:** 1 University of California, Los Angeles, USA; 2 Children’s Hospital of Los Angeles, USA; 3 Yale University, USA; 4 Boston Children’s Hospital, USA; 5 Harvard University, USA; 6 Duke University, USA; 7 University of Washington, USA; 8 Seattle Children’s Research Institute, USA

**Keywords:** aberrant behavior checklist, antipsychotics, autism spectrum disorders, clinical trials, medications, Vineland Adaptive Behavior Scales

## Abstract

Children with autism spectrum disorder are prescribed various medications to address behavior and mood. In clinical trials, individuals taking concomitant psychotropic medications often are excluded to maintain homogeneity and prevent contamination of clinical endpoints. However, this choice may compromise the representativeness of the sample. In a recent study designed to identify biomarkers and endpoints for clinical trials (the Autism Biomarkers Consortium for Clinical Trials), school-age children with autism spectrum disorder were enrolled without excluding for medications, providing the opportunity to examine characteristics of psychotropic medication use and guide future decisions on medication-related inclusion criteria. The aims of the current analysis were (1) to quantify the frequency and type of psychotropic medications reported in school-age children enrolled in the study and (2) to examine behavioral features of children with autism spectrum disorder based on medication classes. Of the 280 children with autism spectrum disorder in the cohort, 42.5% were taking psychotropic medications, with polypharmacy in half. The most commonly reported psychotropic medications included melatonin, stimulants, selective serotonin reuptake inhibitors, alpha agonists, and antipsychotics. Our findings suggest that exclusion of children taking concomitant psychotropic medications could limit the representativeness of the study population, perhaps even excluding children who may most benefit from new treatment options.

**Lay abstract:**

Children with autism spectrum disorder are prescribed a variety of medications that affect the central nervous system (psychotropic medications) to address behavior and mood. In clinical trials, individuals taking concomitant psychotropic medications often are excluded to maintain homogeneity of the sample and prevent contamination of biomarkers or clinical endpoints. However, this choice may significantly diminish the clinical representativeness of the sample. In a recent multisite study designed to identify biomarkers and behavioral endpoints for clinical trials (the Autism Biomarkers Consortium for Clinical Trials), school-age children with autism spectrum disorder were enrolled without excluding for medications, thus providing a unique opportunity to examine characteristics of psychotropic medication use in a research cohort and to guide future decisions on medication-related inclusion criteria. The aims of the current analysis were (1) to quantify the frequency and type of psychotropic medications reported in school-age children enrolled in the ABC-CT and (2) to examine behavioral features of children with autism spectrum disorder based on medication classes. Of the 280 children with autism spectrum disorder in the cohort, 42.5% were taking psychotropic medications, with polypharmacy in half of these children. The most commonly reported psychotropic medications included melatonin, stimulants, selective serotonin reuptake inhibitors, alpha agonists, and antipsychotics. Descriptive analysis showed that children taking antipsychotics displayed a trend toward greater overall impairment. Our findings suggest that exclusion of children taking concomitant psychotropic medications in trials could limit the clinical representativeness of the study population, perhaps even excluding children who may most benefit from new treatment options.

## Introduction

Autism spectrum disorder (ASD) is a neurodevelopmental condition diagnosed in early childhood that is characterized by social communication impairment and the presence of restricted and repetitive patterns of behavior or atypical response to sensory information. It often co-occurs with other neurodevelopmental and psychiatric conditions, including but not limited to social anxiety disorder, attention-deficit hyperactivity disorder (ADHD), and emotional disorders (Simonoff et al., 2008). Comprehensive behavioral interventions are used to target social communication issues and reduce anxiety and aggression within this population. Medications also are widely prescribed to those with ASD to reduce symptoms associated with co-occurring conditions (Esbensen et al., 2009; Jobski et al., 2017; Langworthy-Lam et al., 2002; LeClerc & Easley, 2015; Mahajan et al., 2012; Morgan et al., 2003; Spencer et al., 2013). The Autism Treatment Network (ATN) registry, consisting of 2853 children ages 2–17, reported psychotropic medication use in 27% of children with almost one-third of these children prescribed multiple medications (polypharmacy). Within this cohort, medication use was concentrated in those children with features of (but not necessarily diagnosed with) comorbid psychiatric diagnoses (ADHD, bipolar disorder, obsessive compulsive disorder, depression, or anxiety), with 80% of children in this group receiving some psychotropic medication (Coury et al., 2012). Similar rates of medication use have been reported in other cohorts, both nationally and internationally (Hong et al., 2017; Mandell et al., 2008; Murray et al., 2014; Poling et al., 2017; Spencer et al., 2013; Witwer & Lecavalier, 2005; Ziskind et al., 2020).

In clinical trials, exclusion criteria often constrict the use of concomitant medications (Food and Drug Administration [FDA], 2018; Genre et al., 1999; Van Spall et al., 2007; Yazdani et al., 2020). A systematic review of randomized controlled trials published in medical journals found that 54% of all clinical trials excluded patients due to medication use (Van Spall et al., 2007). There are likely several reasons that concomitant medications are excluded from trials, including minimizing drug-drug interactions (especially with the study drug) and limiting the possibility of medications confounding clinical endpoints, safety endpoints and biomarkers (Brankovic et al., 2019). In clinical trials for ASD, medications that may modulate key neurotransmitter systems with known relevance for neurodevelopment, such as serotonin, dopamine, or glutamate often are excluded (Ghanizadeh & Moghimi-Sarani, 2013; Spanos et al., 2020; Wink et al., 2016). In some studies, children with ASD are required to be completely free of concomitant medication use before they can be enrolled in the trial (Kent et al., 2013; Scahill et al., 2016).

Given the high prevalence of psychotropic medication use in children with ASD, clinical representativeness of cohorts in which medications are excluded may be compromised and undermine the generalizability to the larger ASD population. In order to better understand and characterize psychotropic medication use in a school-age cohort of children with ASD enrolled in a clinical trial readiness study, we surveyed data collected in the Autism Biomarkers Consortium for Clinical Trials (ABC-CT). The ABC-CT was designed to examine brain-based biomarkers and clinical endpoints in school-aged children with ASD for potential application in future clinical trials. Medication use was not excluded. Instead, data were carefully gathered on specific medications and their indications, along with comprehensive behavioral assessments. Our first objective was to quantify the frequency and type of psychotropic medications reported in this school-aged cohort of children with ASD enrolled in the ABC-CT. Our second objective was to examine the clinical features associated with the use of psychotropic medications and to describe behavioral profiles by medication subtype.

## Methods

### Data source

Data were provided by the ABC-CT, a longitudinal, multisite study focused on assessing the reliability and scalability of electroencephalogram (EEG) and eye-tracking biomarkers of social function in children aged 6–11 years with ASD and an age- and sex-matched typically developing comparison cohort. The ABC-CT did not exclude for concomitant medications but did require that the medication regimen remained stable for at least 8 weeks prior to enrollment. Inclusion criteria included age range of 6–11 years, cognitive ability of 60–150 as assessed by the Differential Ability Scales (DAS), 2nd Edition (DAS-II; Elliot 2007), and clinical ASD diagnosis (McPartland et al., 2020). Exclusionary criteria included: known genetic or neurological syndrome with an established link to autism, history of epilepsy or current seizures, motor or sensory impairment that would interfere with the valid completion of study measures, history of significant prenatal or perinatal birth injury or neonatal brain damage, or the presence of any known severe environmental circumstances such as nutritional or psychological deprivation. Participants were recruited at five sites across the United States to gather geographically representative data. Data regarding medication use were gathered via caregiver interview at each time point.

Participants took part in three study visits (Time 1: Baseline; Time 2: 6 weeks after baseline; and Time 3: 24 weeks after baseline). At each time point, children completed clinical/behavioral, EEG, and eye-tracking assessments. Parents provided interview and questionnaire reporting of their child’s behavior and medical and intervention history. Caregiver questionnaires included the aberrant behavior checklist (Aman et al., 1985), the Autism Impact Measure (Kanne et al., 2014), the Pervasive Developmental Disorder Behavior Inventory (Cohen & Sudhalter, 2005), and the Social Responsiveness Scale, 2nd Edition (Constantino & Gruber, 2012). Data collection was standardized across all five sites in accordance with detailed manuals of procedures in order to limit the potential for site effects. Here, we used a cross-sectional approach and focused on Time 1 data to examine medication use at enrollment. Time 1 data collection took place from December 2015 to March 2016. While there was involvement from members of the ASD community in the formulation and completion of the ABC-CT, there was no direct community involvement in the design or analyses presented within this manuscript.

### Participants

*N* = 280 children with ASD were enrolled in the ABC-CT study across the five participating sites: Boston Children’s Hospital, Duke University, the University of California Los Angeles (UCLA), University of Washington, and Yale University. The majority of the sample was male (*n* *=* 215), White (*n* = 190), and from families whose median household income (*Mdn* *=* $100,001–$150,000) was above the national median (National *Mdn* *=* $68,703; U.S. Census Bureau, 2021). Although the sample was disproportionately male (77% male; 23% female), the 3:1 male-to-female sex ratio corresponds to sex ratios identified within recent prevalence studies (Loomes et al., 2017). Further demographic information is presented in Table 1.

**Table 1.** Participant demographics.

Demographics	Descriptive
Participant age	*M* *=* 8.6 years, *SD* *=* 1.7, range = 6.0–11.6 years
Participant sex	Male: 77.0% Female: 23.0%
Participant race	White: 67.8% Mixed race: 16.1% African-American/Black: 7.9% Asian-American: 5.4% American Indian or Alaska Native: 0.7% Other: 2.1%
Participant ethnicity	Not Hispanic or Latino: 81.4% Hispanic or Latino: 18.6%
ADOS Calibrated Severity Score	*M* *=* 7.7, *SD* *=* 1.8, range = 4.0–10.0
Verbal IQ	*M* *=* 96.0, *SD* *=* 20.7, range = 37.0–152.0
Nonverbal IQ	*M* *=* 97.5, *SD* *=* 16.9, range = 57.0–164.0
Full scale IQ	*M* = 96.6, *SD* *=* 18.1, range = 60.0–150.0
Household income	$0–25,000: 6.7% $25,001–50,000: 15.1% $50,001–75,000: 11.4% $75,001–100,000: 13.9% $100,001–150,000: 23.6% $150,000+: 26.8% Missing: 2.5%
Highest parent educational level	High school degree: 3.6% Some college or associate degree: 22.5% Bachelor’s degree: 29.6% Graduate degree: 44.3%

*SD*: standard deviation ; ADOS: Autism Diagnostic Observation Schedule.

### Measures

Participants took part in an extensive battery of behavioral assessments within the ABC-CT study. Based on our clinical constructs of interest, we selected the following measures for analysis: medication use interview, the DAS-II, the Vineland Adaptive Behavior Scale-II (VABS-II), and the Aberrant Behavior Checklist, 2nd Edition (ABC-2).

#### DAS-II

The DAS-II (Elliott, 2007) is a direct assessment of cognitive ability in children aged 2–17 years that yields verbal (VIQ) and nonverbal (NVIQ) ability scores. Depending on the chronological age and developmental ability of the child within the ABC-CT study, either the DAS-II Early Years (2:6–6:11 years) or DAS-II School-Age (7:0–17:11 years) battery was administered. If deviation intelligence quotient (IQ) scores, which measure the difference between each individual score and the mean score, could not be calculated for verbal or nonverbal IQ, then ratio IQ scores were used. VIQ and NVIQ scores were reported as standard scores with a mean of 100 and a standard deviation of 15. Scores on each of these measures were classified as “borderline clinically significant” if they fell between one and two standard deviations below the mean. Scores that fell beyond two standard deviations below the mean were classified as “clinically significant.”

#### VABS-III

The VABS-III (Sparrow et al., 2016) is a semi-structured parent interview that measures four domains of adaptive functioning: communication, daily living skills, socialization, and motor skills. For the ABC-CT study, only the communication, daily living skills, and socialization domain items were administered. Standard scores for the three VABS-III domains as well as the overall Adaptive Behavior Composite (ABC) score were computed, with higher scores indicating greater adaptive ability. As the ABC is calculated from the three abovementioned domain scores and provides a multimodal summary of adaptive functioning, only the ABC score was used in the analyses. VABS: ABC scores were reported as standard scores with a mean of 100 and a standard deviation of 15. Scores were classified as “borderline clinically significant” if they fell between one and two standard deviations below the mean. Scores that fell beyond two standard deviations below the mean were classified as “clinically significant.”

#### ABC-2

The ABC-2 (Aman & Singh, 2017) is a proxy-report measure of challenging behaviors commonly experienced by children with neurodevelopmental disorders, with higher scores reflecting greater severity of challenging behaviors. Parents completed the ABC-2 for their child. While the ABC-2 is comprised of five behavioral subscales—irritability, social withdrawal, stereotypic behavior, hyperactivity/noncompliance, and inappropriate speech—only subdomain scores for the irritability and hyperactivity/noncompliance domains were used in the analyses of this article, as these challenging behaviors are most often identified within comorbidities in ASD (e.g. insomnia, inattention/hyperactivity, and anxiety). For the two ABC-2 domains of hyperactivity and irritability, scores were reported as *Z*-scores, with a mean of 0 and standard deviation of 1. Clinically significant scores on the ABC-2 were described as “clinically elevated” to remain consistent with the terminology set forth by the ABC-2 guidelines for clinical findings. As higher scores indicate greater severity of challenging behaviors, scores were classified as “borderline clinically elevated” if they fell between one and two standard deviations above the mean, while scores beyond two standard deviations above the mean were classified as “clinically elevated.”

#### Medication

Derived from parent-report of their child’s medication use, both the number and type of medications used at Time 1 were recorded for each participant. Medication type was first broadly defined within a dichotomous coding system based on whether the medication did or did not have an effect on the central nervous system (psychotropic vs non-psychotropic medication). A more detailed examination of the psychotropic medication category was then conducted. Sixteen distinct medication classes were created to characterize psychotropic medication use as follows: (1) alpha agonist, (2) SSRI, (3) stimulant, (4) antipsychotic, (5) antiepileptic, (6) selective norepinephrine, (7) dopamine/norepinephrine, (8) NMDA antagonist, (9) 5-HT agonist, (10) melatonin, (11) oxytocin, (12) Benadryl, (13) headache medication, (14) benzodiazepine, (15) GABA Supplement, and (16) other which included dimethylethanolamine (DMAE). Non-psychotropic medication included most antihistamines, nonsteroidal anti-inflammatory drugs, and other non-psychoactive medications.

### Analyses

#### Demographic and clinical features of children taking psychotropic medications

The number of participants taking psychotropic, non-psychotropic, and no medications were calculated. For the sake of analysis, two groups were created: a psychotropic medication group and a comparison group, which included participants taking only non-psychotropic medications and participants not taking any medications at all.

All analyses were run in IBM SPSS Statistics (Version 27.0). An independent *t-*test was run to compare groups on the variable of child age and a chi-square test of independence compared groups on the variable of child sex. Five independent *t-*tests compared groups on the variables of DAS-2 VIQ, DAS-2 NVIQ, VABS-III ABC, ABC-2 Hyperactivity, and ABC-2 Irritability. Adjustments for multiple comparisons were made using Bonferroni correction, such that significance thresholds for each test were set at *p* < .007 (determined by *p* < .05/7 tests). For chi-square tests with 1 degree of freedom, magnitudes of effect sizes as measured by Cramer’s V correspond to the following values: .1 for a small effect, .3 for a medium effect, and .5 for a large effect (Cohen, 1988). The magnitudes of effect sizes for *t-*tests, as measured by Cohen’s *d*, correspond to the following values: .2 for a small effect, .5 for a medium effect, and .8 for a large effect (Cohen, 1988).

**Figure 1.**
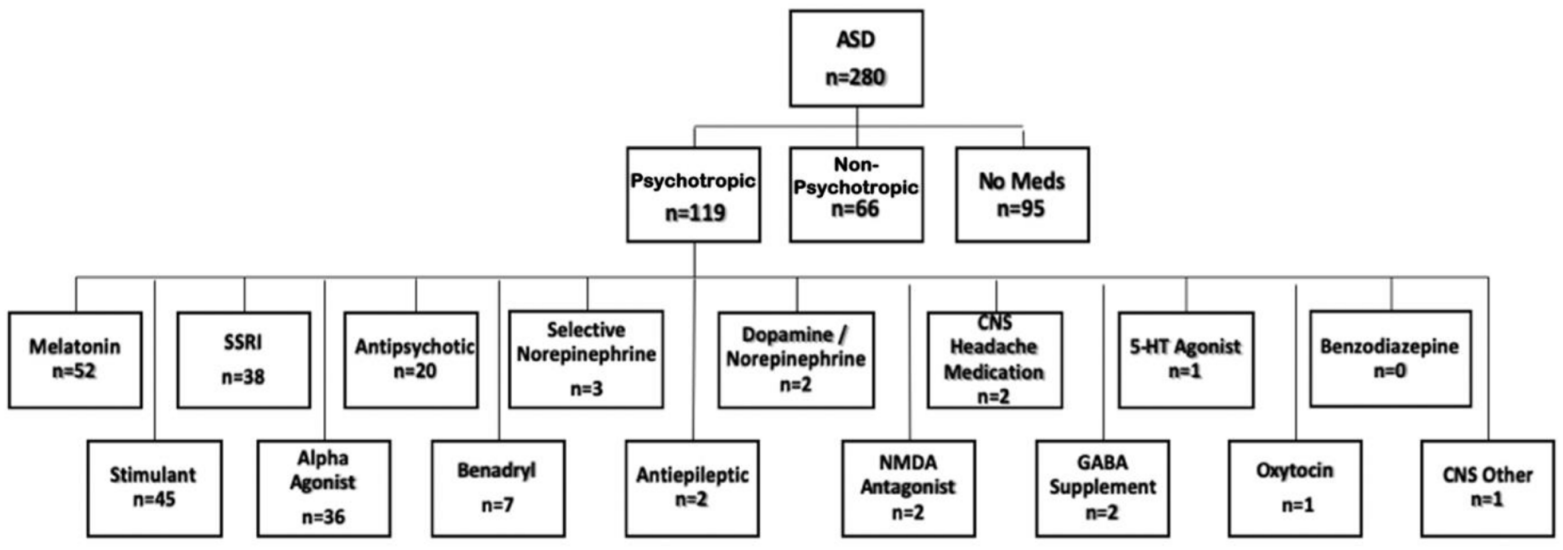
Number of participants with reported medication use by category and class. The psychotropic group (*n* = 119) was composed of participants who used one or more psychotropic medication while the non-psychotropic (*n* = 66) and no meds (*n* = 95) groups encompassed those who did not use any psychotropic medication.

Among the participants who reported psychotropic medication use, the most commonly endorsed psychotropic medication classes were melatonin (*n* = 53), stimulants (*n* = 45), SSRIs (*n* = 37), alpha agonists (*n* = 36), and antipsychotics (*n* = 20) (Figure 1).

#### Demographic and clinical features associated with mono- and polypharmacy within the psychotropic medication group

Because the parents of these children reported all medications taken at Time 1, monopharmacy (i.e. taking one medication) and polypharmacy (i.e. taking more than one medication, either within or across medication classes) subgroups were created for participants on psychotropic medication. Only one participant assigned to the polypharmacy subgroup was taking more than one medication only within a single psychotropic medication class. This participant was taking two antipsychotic medications. All others were taking medications across two or more psychotropic medication classes. For the polypharmacy subgroup, the range in number of psychotropic medication classes endorsed was calculated, as were rates of co-prescription for melatonin, stimulants, SSRIs, alpha agonists, and antipsychotics. Sex ratios as well as means and standard deviations for the variables of child age, DAS-II VIQ, DAS-II NVIQ, VABS-III ABC, ABC-2 Hyperactivity, and ABC-2 Irritability scores were computed for the monopharmacy and polypharmacy subgroups. The proportion of borderline and clinically significant scores on all clinical variables was assessed for the monopharmacy and polypharmacy subgroups.

#### Behavioral profiles of most highly endorsed psychotropic medication classes

Scores of cognition, adaptive skills, and aberrant behaviors were summarized for melatonin, stimulants, SSRIs, alpha agonists, and antipsychotics. Given that the number of participants with reported current medication use within each psychotropic medication class was not mutually exclusive, the behavioral profiles of the overall ASD cohort and each of the five most highly endorsed psychotropic medication classes were examined descriptively. Sex ratios as well as means and standard deviations for the variables of child age, VIQ, NVIQ, VABS-III ABC, ABC-2 Hyperactivity, and ABC-2 Irritability scores were computed for each class. Furthermore, the proportion of clinically significant scores on all abovementioned variables except for child age and sex was assessed in accordance with measurement scoring standards.

## Results

### Demographic and clinical features of children taking psychotropic medications

Of the 280 ASD participants, the majority (*n* = 185; 74.0%) reported taking medications, with *n* *=* 119 (47.6%) participants on psychotropic medication, *n* = 66 (26.4%) participants on non-psychotropic medication, and *n* = 95 participants on no medication. The non-psychotropic medication group and the no medication group were combined to form the “comparison group.”

Participants within the psychotropic medication group were older than participants in the comparison group, *t*(278) = 5.44, *p* < .001, *d* = .66. There were no significant sex differences between groups, with 79.0% of the psychotropic medication group and 75.2% of the comparison group being male, χ^2^(1) = .57, *p* = .45, Cramer’s V = .05.

Groups did not differ significantly on mean VIQ scores, *t*(278) = 1.96, *p* = .05, *d* = .24, nor on mean NVIQ scores, *t*(278) = .04, *p* = .97, *d* = .005. Groups also did not differ significantly on mean VABS-III ABC scores after adjusting for multiple comparisons, *t*(277) = 2.34, *p* = .02, *d* = .28. Finally, for the variables measuring challenging behaviors, namely hyperactivity and irritability, the groups did not differ significantly on mean ABC-2 Hyperactivity scores, *t*(274) = 2.16, *p* = .03, *d* = .26. The psychotropic medication group did have a significantly higher mean ABC-2 Irritability score compared to the comparison group, *t*(274) = 3.04, *p* = .003, *d* = .37. See Table 2 for means and standard deviations for child age and each of the clinical variables split by group.

**Table 2.** Means, standards deviations, and ranges for child age and the clinical variables assessing cognition, adaptive functioning, and challenging behaviors for the psychotropic medication and comparison groups.

Variable	Psychotropic medication group (*n* = 119)			Comparison group (*n* = 161)		
	Mean	*SD*	Range	Mean	*SD*	Range
Age	9.14	1.58	(6.04–11.55)	8.11	1.56	(6.01–11.48)
DAS-II VIQ	98.75	19.51	(55–141)	93.88	21.34	(37–152)
DAS-II NVIQ	97.56	15.60	(64–147)	97.48	17.86	(57–164)
VABS-III ABC	70.89	13.46	(32–98)	74.84	14.28	(29.5–114)
ABC-2 Hyperactivity	0.47	0.88	(–1.19–2.95)	0.23	0.91	(–1.26–2.72)
ABC-2 Irritability	0.66	1.18	(–1.19–5.06)	0.25	1.09	(–1.21–5.06)

*SD*: standard deviation; DAS-II: Differential Ability Scale, 2nd edition; VIQ: verbal IQ; NVIQ: non-verbal IQ; VABS-III: Vineland Adaptive Behavior Scale-III; ABC-2: Aberrant Behavior Checklist, 2nd Edition.

Please note that ABC-2 Hyperactivity and ABC-2 Irritability mean scores are presented as *Z*-scores.

### Demographic and clinical features associated with mono- and polypharmacy

Of the *n* = 119 participants with reported psychotropic medication use, 50.4% (*n* = 60) of participants were taking one medication, while 49.6% (*n* = 59) of participants were taking more than one medication, within two or more psychotropic medication classes (i.e. polypharmacy). Within the polypharmacy subgroup (*n* = 59), 42.4% (*n* *=* 25) were taking medications from two classes, 37.3% (*n* *=* 22) from 3 classes, and 20.3% (*n* = 12) from ⩾4 classes. Rates of co-prescription with other psychotropic medication classes for the five most commonly endorsed psychotropic medications were as follows: melatonin (52.8%), stimulants (80%), SSRIs (91.9%), alpha agonists (91.7%), and antipsychotics (90%). Interestingly, SSRIs and alpha agonists were most frequently co-prescribed (*n* = 16), followed by stimulants and melatonin (*n* = 15), and stimulants and SSRIs (*n* = 13).

The majority of participants within both mono- and polypharmacy subgroups were male; however, a larger proportion of females was found for the polypharmacy subgroup compared to the monopharmacy subgroup (Table 3).

**Table 3.** Count and percentages of males and females within the monopharmacy and polypharmacy subgroups.

Group	Male		Female	
	Count	Percentage	Count	Percentage
Monopharmacy (*n* *=* 60)	51	85.0%	9	15.0%
Polypharmacy (*n* *=* 59)	43	72.9%	16	27.1%

**Figure 2.**
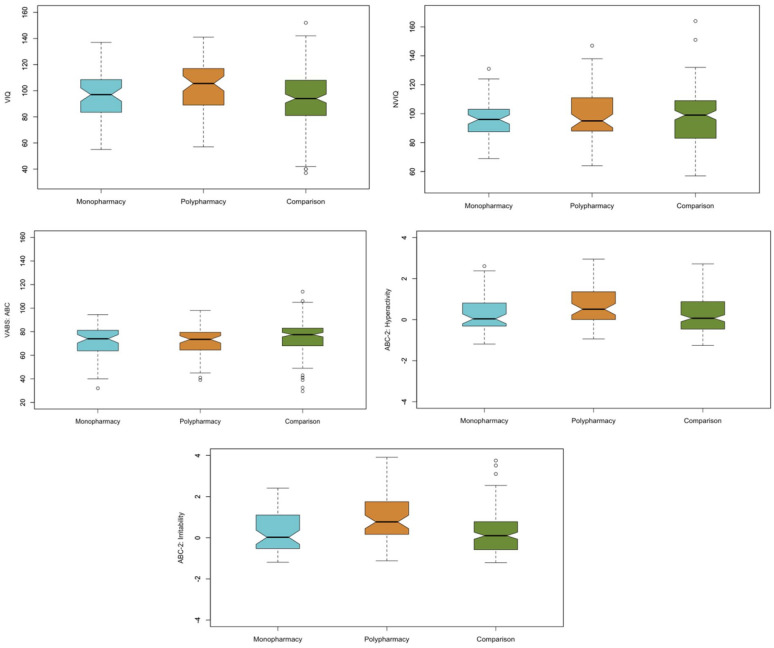
Boxplots depicting the monopharmacy and polypharmacy subgroups as well as the non-psychotropic and no medication “comparison group” on the clinical measures of VIQ, NVIQ, VABS ABC, ABC-2 Hyperactivity, and ABC-2 Irritability. Upper and lower limits of each boxplot represent the 75th and 25th percentile of scores, respectively. Whiskers extending beyond the box are 1.5 times the interquartile range, within which the maximum and minimum scores are included. Median scores are indicated by the solid black line, and notches represent the 95% confidence interval around each median score. Outliers are represented by open circles, which indicate the level of the Y-axis that is beyond 1.5× the interquartile range. Outliers were not excluded from the analysis.

Participants in the polypharmacy subgroup were, on average, older than those in the monopharmacy subgroup (Table 4). The polypharmacy subgroup had higher mean VIQ and NVIQ scores than the monopharmacy subgroup and also showed a greater degree of challenging behaviors (based on higher mean ABC-2 Hyperactivity and Irritability domain scores) compared to their monopharmacy counterparts. There were no qualitative differences in ABC between groups. See Figure 2 for further visualization of subgroup distributions on all clinical measures.

**Table 4.** Clinical characteristics of the monopharmacy and polypharmacy subgroups.

Variable	Monopharmacy (*n* = 60)			Polypharmacy (*n* = 59)		
	Mean	*SD*	Range	Mean	*SD*	Range
Age	8.71	1.63	(6.04–11.55)	9.58	1.42	(6.43–11.51)
DAS-II VIQ	94.72	17.98	(55–137)	102.85	20.28	(57–141)
DAS-II NVIQ	95.70	12.56	(69–131)	99.46	18.03	(64–147)
VABS-III ABC	70.74	14.32	(32–94.5)	71.32	12.64	(39–98)
ABC-2 Hyperactivity	0.28	0.89	(–1.19–2.61)	0.66	0.84	(–0.94–2.95)
ABC-2 Irritability	0.38	1.17	(–1.19–5.06)	0.94	1.14	(–1.12–3.91)

*SD*: standard deviation; DAS-II: Differential Ability Scale, 2nd edition; VIQ: verbal IQ; NVIQ: non-verbal IQ; VABS-III: Vineland Adaptive Behavior Scale-III; ABC-2: Aberrant Behavior Checklist, 2nd Edition.

**Figure 3.**
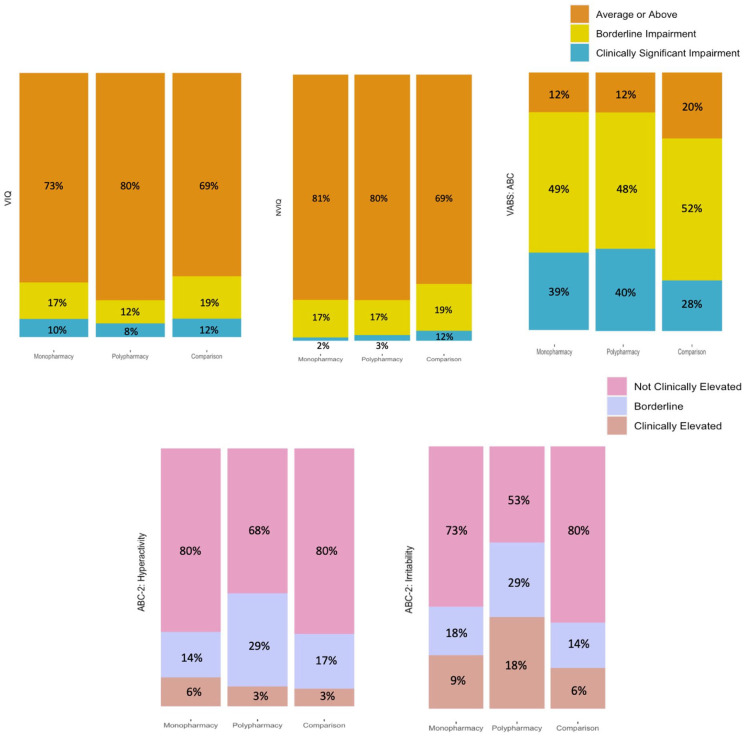
Stacked bar charts depicting the monopharmacy and polypharmacy subgroups as well as the non-psychotropic and no medication “comparison group” for each of the five clinical measures (VIQ, NVIQ, VABS: ABC, ABC-2: Hyperactivity, and ABC-2: Irritability). Percentages of scores that fall within the “Average of Above”/“Not Clinically Elevated,” “Borderline Impairment”/“Borderline,” and “Clinically Significant Impairment”/“Clinically Elevated” ranges are provided.

With regard to clinical cut offs, the monopharmacy and polypharmacy subgroups demonstrated similar patterns in NVIQ and adaptive functioning (Figure 3). In addition, both subgroups showed greater challenges in irritability compared to hyperactivity, as marked by a higher proportion of “clinically elevated” scores in the ABC-2 Irritability domain compared to the ABC-2 Hyperactivity domain. The subgroups diverged when examining VIQ and the actual proportions of “not clinically elevated,” “borderline,” and “clinically elevated” scores on the ABC-2 domains. While the majority of VIQ scores fell within the “average or above” range for both subgroups, the monopharmacy subgroup had a higher proportion of scores within the combined “borderline” and “clinically significant” impairment range than the polypharmacy subgroup. However, on the ABC-2 domains of Hyperactivity and Irritability, the polypharmacy subgroup evidenced a greater degree of severe challenging behaviors, as indicated by their considerably higher proportion of combined “borderline” and “clinically elevated” scores on both domains, compared to the monopharmacy subgroup.

### Behavioral profiles of most highly endorsed psychotropic medication classes

Comparing the five most highly endorsed psychotropic medication classes (melatonin, stimulants, SSRIs, alpha agonists, and antipsychotics) with the total number of ASD participants (All ASD) and ASD participants that endorsed psychotropic medication use, the majority across all groups was male, with percentages ranging from 75.0% male within the antipsychotics class to 88.9% male within the alpha agonists class (Table 5).

**Table 5.** Count and percentages of males and females within the All ASD cohort, ASD participants on psychotropic medication, and the five most highly endorsed CNS medications classes.

Group	Male		Female	
	Count	Percentage	Count	Percentage
All ASD (*N* = 280)	215	76.8%	65	23.2%
Psychotropic (*N* = 119)	94	79.0%	25	21.0%
Melatonin (*n* *=* 53)	43	81.1%	10	18.9%
Stimulants (*n* *=* 45)	34	75.6%	11	24.4%
SSRIs (*n* *=* 37)	28	75.7%	9	24.3%
Alpha agonists (*n* *=* 36)	32	88.9%	4	11.1%
Antipsychotics (*n* *=* 20)	15	75.0%	5	25.0%

ASD: autism spectrum disorder.

Furthermore, the All ASD cohort were, on average, younger than those who reported use of psychotropic medications and those that specifically reported use of melatonin, stimulants, SSRIs, alpha agonists, or antipsychotics (Table 6).

**Table 6.** Clinical characteristics by CNS medication class and the All ASD cohort. All scores Mean (SD).

	All ASD (*N* = 280)	Psychotropic (*n* = 119)	Melatonin (*n* = 53)	Stimulants (*n* = 45)	SSRIs (*n* = 37)	Alpha agonists (*n* = 36)	Antipsychotics (*n* = 20)
Age	8.55 (1.65)	9.14 (1.58)	8.79 (1.51)	9.57 (1.53)	9.55 (1.47)	9.73 (1.37)	9.47 (1.61)
VIQ	95.95 (20.69)	98.75 (19.51)	98.13 (19.86)	105.64 (16.69)	104.05 (22.15)	101.00 (18.99)	88.10 (15.49)
NVIQ	97.52 (16.91)	97.56 (15.60)	99.15 (15.93)	99.00 (15.87)	100.51 (17.37)	100.36 (18.97)	88.35 (8.41)
VABS: ABC	73.17 (14.05)	70.89 (13.46)	70.81 (13.87)	73.04 (12.04)	71.49 (12.37)	70.72 (15.32)	60.92 (13.84)
ABC-2: Hyperactivity	0.33 (0.91)	0.47 (0.88)	0.41 (0.84)	0.62 (0.83)	0.52 (0.90)	0.62 (0.85)	0.85 (0.87)
ABC-2: Irritability	0.42 (1.14)	0.66 (1.18)	0.65 (1.11)	0.54 (1.07)	0.82 (1.24)	0.94 (0.94)	1.53 (1.37)

ASD: autism spectrum disorder; VIQ: verbal IQ; NVIQ: non-verbal IQ; VABS-III: Vineland Adaptive Behavior Scale-III; ABC-2: Aberrant Behavior Checklist, 2nd Edition.

**Figure 4.**
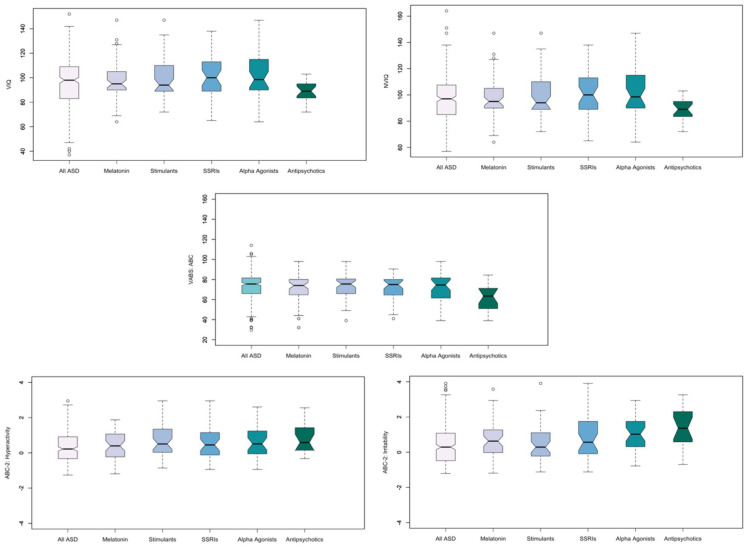
Boxplots depicting the five most highly endorsed psychotropic medication classes (melatonin, stimulants, SSRIs, alpha agonists, and antipsychotics) and the All ASD cohort on the clinical measures of VIQ, NVIQ, VABS ABC, ABC-2 Hyperactivity, and ABC-2 Irritability. Upper and lower limits of each boxplot represent the 75th and 25th percentile of scores, respectively. Whiskers extending beyond the box are 1.5 times the interquartile range, within which the maximum and minimum scores are included. Median scores are indicated by the solid black line, and notches represent the 95% confidence interval around each median score. Outliers are represented by open circles, which indicate the level of the Y-axis that is beyond 1.5 times the interquartile range. Outliers were not excluded from the analysis.

While use of melatonin, stimulants, SSRIs, or alpha agonists had trends of higher VIQ and NVIQ scores than those of the All ASD cohort, there were also trends in higher hyperactivity and irritability scores and lower adaptive functioning. Although this examination was descriptive, children who reported use of antipsychotics showed a trend toward greater overall impairment than both the All ASD cohort and the other psychotropic medication groups, as evidenced by lower mean VIQ, NVIQ, and adaptive functioning scores and by higher hyperactivity and irritability scores. See Figure 4 for visualization of group distributions on all clinical measures.

While the sample size of children taking antipsychotics was considerably smaller than those of the other psychotropic medication classes, it is important to note that for many of the measures, and especially for VIQ and NVIQ, the variation in the antipsychotics group was smaller (Figure 4), suggesting greater homogeneity in NVIQ within the antipsychotics class.

**Figure 5.**
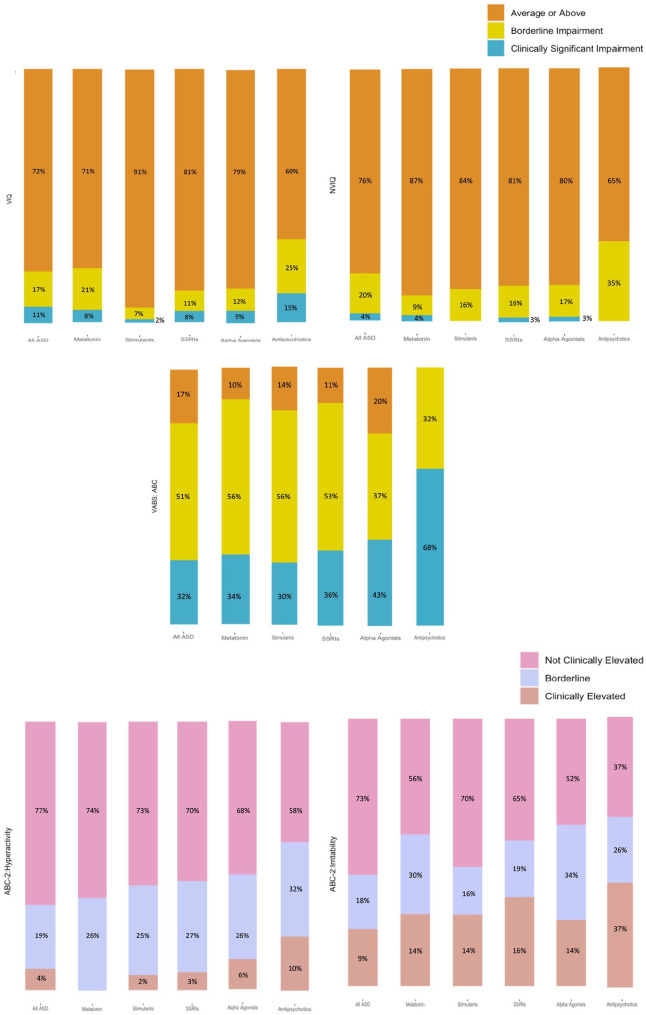
Stacked bar charts for each of the five clinical measures (VIQ, NVIQ, VABS: ABC, ABC-2: Hyperactivity, ABC-2: Irritability). Percentages of scores that fall within the “Average of Above”/“Not Clinically Elevated,” “Borderline Impairment”/“Borderline,” and “Clinically Significant Impairment”/“Clinically Elevated” ranges are provided for each measure and for each group (All ASD cohort, melatonin, stimulants, SSRIs, alpha agonists, and antipsychotics).

In terms of clinical significance of scores, the antipsychotic class showed qualitatively higher percentage of scores falling within the “borderline clinically significant” and “clinically significant” range for each of the five clinical measures compared to both the All ASD cohort and the other four psychotropic medication classes (Figure 5). Across all five clinical measures, adaptive functioning, as measured by VABS: ABC scores, was the most impaired (i.e. highest proportion of borderline and clinically elevated scores) across groups.

## Discussion

The Autism Biomarkers Consortium for Clinical Trials (ABC-CT) provided an opportunity to examine medication use patterns in a geographically diverse cohort of school-age children with ASD engaged in a study that required in-person assessments and longitudinal engagement in research. We characterized psychotropic medication use in 280 children with ASD between the ages of 6 and 11 years and began to identify clinical patterns based on medication type. Our results are consistent with previous studies showing that individuals with ASD have a high rate of medication use, likely to target co-occurring neurobehavioral and psychiatric symptoms or diagnoses (Esbensen et al., 2009; Spencer et al., 2013; Langworthy-Lam et al., 2002). Most of our analyses were descriptive, in part due to the heterogeneity of the sample and in part to fulfill our goal of leveraging this rich data set to better understand medication use in order to generate hypotheses that could be tested in future studies. Some themes and trends emerged that warrant further consideration. First, psychotropic medications were used in almost 50% of children, with polypharmacy occurring in half of these children. This rate is higher than those reported in large clinically ascertained cohorts of children with ASD. Second, there was a range of medication targets based on the top-five categories, with medications targeting insomnia (melatonin and certain alpha agonists), ADHD (alpha agonists and stimulants), mood disturbances (SSRIs), and behavioral challenges (antipsychotics). We did not ask parents about the indication for medication use, however, so these co-occurring conditions and treatment targets are inferred. Third, irritability was endorsed more for children taking medications, suggesting that either the medications did not adequately address these challenges or that even with some relief through pharmacotherapy, this symptom requires more aggressive management. Fourth, polypharmacy was common, with half of the psychotropic medication group taking medications from multiple medication classes. Parents of these children endorsed an even higher rate of hyperactivity and irritability, suggesting medication resistance to these co-occurring conditions, inadequate prescribing practices for these symptoms, inadequacy of these measures to truly assess change with pharmacotherapy, or that these children had more severe symptomatology to begin with that has yet to be substantially reduced by medication use. Finally, upon examination of clinical features based on medication class, those taking antipsychotics tended to show the most clinical impairment. The trajectory of these co-occurring conditions with treatment was not available, which would have been helpful to determine the clinical course and impact of the medications on clinical symptoms.

Currently, the only FDA-approved medications for ASD are antipsychotics (risperidone and ariprazole) for the indication of irritability (Accordino et al., 2016; Alfageh et al., 2020; Fallah et al., 2019; McCracken et al., 2002; Marcus et al., 2009), but children with ASD are prescribed psychotropic medications widely, as evidenced by the ATN data showing psychotropic medication use in 27% of children ages 2–17, with rates increasing with age, and highest rates in later school age and adolescence. Why was medication use even higher in this research cohort? There are several possible reasons that may, in fact, more accurately represent the characteristics of children with ASD enrolled in clinical trials. First, most of the participants in this cohort were white, more than half of the participants had a household income of more than $100,000, and more than half had parents with bachelor’s or graduate degrees. In the ATN study, insurance status served as a proxy for socioeconomic status, and investigators found that children with private insurance had a higher rate of psychotropic medication use than those with other or no insurance. This study also found that non-white and Latino children had lower use of psychotropic medications than white/non-Latino children (Coury et al., 2012). The factors driving the disparity in medication prescribing practices likely are complex, involving differences in access to diagnosis and care, cultural influences (and stigma) on perception of psychopharmacology and on co-occurring neuropsychiatric diagnoses, and even practitioner prescribing practices. Engagement in research, particularly studies involving frequent in-person assessments, requires financial resources and time that can lead to an inherent enrollment bias, and these same factors may contribute to the high rate of psychotropic medication use. These factors must be appreciated when clinical trials are being designed, particularly if psychotropic medication use is excluded.

From a clinical standpoint, parents of children taking psychotropic medications endorsed more irritability. Overall, this finding is consistent with studies that show that children with ASD taking psychotropic medications have higher levels of clinical morbidity (as defined by measures such as the CGI-Severity of Illness Scores; Shekunov et al., 2017). Of note, in this cohort, *Diagnostic and Statistical Manual of Mental Disorders, Fifth Edition* (*DSM*-5) diagnoses of psychiatric comorbidities were not made, nor were data available on symptom severity prior to medication use. The specific indication for the medication was not recorded nor were data on the time course of medication use and symptoms. Studies that can ascertain this level of detail in the medical history may guide decision-making around inclusion of participants based on psychotropic medication use.

The most commonly reported psychotropic medications were melatonin, stimulants, SSRI’s, alpha agonists, and antipsychotics. The high rate of melatonin use is consistent with the high prevalence of insomnia in ASD, with rates as high as 80% of children with ASD (Williams Buckley et al., 2020). Cohort studies and clinical trials of melatonin in ASD have shown effectiveness for sleep latency and duration (Gringras et al., 2017; Rossignol & Frye, 2011; for review, see Rzepka-Migut, 2020). Furthermore, melatonin does not require a prescription, thus improving access and likely facilitating its use. With regard to implications for clinical trials, one could posit that medication use itself indicates specific clinical subgroups within the autism spectrum. Our efforts to qualitatively compare behavioral profiles between medication classes aimed to identify such subgroups. The most notable finding was that the group taking antipsychotics showed trends toward more clinical impairment across domains (cognitive, adaptive skills, and aberrant behaviors). Inclusion of children taking this class of medications may, therefore, introduce a clinical cohort with greater symptom severity and/or psychiatric comorbidities. It should be noted that polypharmacy, which was present in half of those participants taking psychotropic medications, precluded a clean comparison of mutually exclusive groups (meaning those only taking one class of medication). However, given the high rate of polypharmacy, such a comparison may prove difficult even in a larger clinical cohort.

Limitations to this analysis included the lack of data on the specific indication and exact dosage of medications, lack of medical history data that queried the effect of these medications on symptoms, and limited information on the perceived effectiveness of these medications on target behaviors or co-occurring conditions either by clinician or caregiver. In addition, as the primary aims of the ABC-CT were to produce a large-scale analysis on biomarkers in children with ASD, and the majority of enrollment was completed at major medical centers, the sample is not completely representative of the ASD population at large.

### Clinical significance

This study highlights the high prevalence of psychotropic medication use in children with ASD, particularly in the demographic of children enrolled in research studies. The fact that children from white and high socioeconomic status (SES) backgrounds tend to show higher rates of medication use suggests that there are complex social determinants of prescription practices. Only atypical antipsychotics are FDA approved for an autism-specific indication (irritability), but children with ASD are widely being prescribed medications, not for core symptoms, for a variety of co-occurring conditions that significantly impact quality of life, such as insomnia, inattention, impulsivity, hyperactivity, and mood disturbances. Studies also have shown that the use of complementary/alternative medications is highly prevalent in ASD (Volkmar et al., 2014). Clinicians (both primary care and specialists) caring for patients with ASD should ask parents about all types of treatments, both prescribed and over-the-counter, and they should consider the potential impact of these treatments on behavior and development. Each medication has the potential for side effects that can influence a child’s current presentation. Moreover, clinicians should caution against polypharmacy, as drug–drug interactions can further exacerbate a child’s functioning. Ideally, for every medication prescribed, there should be a well-defined clinical target and a method for measuring success.

## Conclusion

This study compels us to find informed ways to examine clinical endpoints and biomarkers should we choose to include these children in studies and trials, with the ultimate goal of maximizing clinical representativeness of cohorts. This clinical trial readiness study is well positioned to compare putative biomarkers, such as resting state EEG power and connectivity or eye-tracking measures of social attention, between and across medication classes to determine if these psychotropic medications alter signal in a meaningful or confounding way and to develop algorithms to account for the effect of these medications on these biomarkers. These analyses will directly inform choices about inclusion or exclusion of participants in future clinical trials in ASD.
